# Social franchising of community‐based HIV testing and linkage to HIV care and treatment services: an evaluation of a pilot study in Tshwane, South Africa

**DOI:** 10.1002/jia2.25216

**Published:** 2018-12-20

**Authors:** Simukai Shamu, Thato Farirai, Locadiah Kuwanda, Jean Slabbert, Geoffrey Guloba, Suzanne Johnson, Sikhulile Khupakonke, Nomea Masihleho, Julius Kamera, Nkhensani Nkhwashu

**Affiliations:** ^1^ Foundation for Professional Development Pretoria South Africa; ^2^ School of Public Health University of the Witwatersrand Johannesburg South Africa; ^3^ USAID Pretoria South Africa

**Keywords:** social franchising, HIV testing, HIV positivity, linkage to care, HIV testing and linkage cost, South Africa

## Abstract

**Introduction:**

Although HIV testing services (HTS) have been successfully task‐shifted to lay counsellors, no model has tested the franchising of HTS to lay counsellors as independent small‐scale business owners. This paper evaluates the effectiveness of a social franchisee (SF) HTS‐managed pilot project compared to the Foundation for Professional Development (FPD) employee‐managed HTS programme in testing and linking clients to care.

**Methods:**

Unemployed, formally employed or own business individuals were engaged as franchisees, trained and supported to deliver HTS services under a common brand in high HIV‐prevalent communities in Tshwane district between 2016 and 2017. SFs were remunerated per‐HIV test and received larger payments per‐HIV‐positive client linked to care. In the standard HTS, FPD employed counsellors received similar training and observed similar standards as in the SF HTS, but were remunerated through the normal payroll. We assessed the proportion of clients tested, HIV positivity, linkage to care and per‐counsellor cost of HIV test and linkage to care in the two HTS groups.

**Results:**

The SF HTS had 19 HIV counsellors while FPD HTS employed 20. A combined total of 84,556 clients were tested by SFs (50.5%: 95% confidence interval (CI) 50.2 to 50.8)) and FPD (49.5%: 49.2 to 49.8). SFs tested more females than FPD (54.1%: 53.6 to 54.6 vs. 48%: 47.7 to 48.7). SFs identified more first‐time testers than FPD (21.5%: 21.1 to 21.9 vs. 8.9%: 8.6 to 9.1). Overall, 8%: 7.9 to 8.2 tested positive with more clients testing positive in the SF (10.2%: 9.9 to 10.5) than FPD (5.9%: 5.6 to 6.1) group. The SFs identified more female HIV‐positive clients (11.1%: 10.7 to 11.6) than FPD (6.5%: 6.2 to 6.9). The SFs linked fewer clients to HIV care and treatment (60.0%: 58.5 to 61.5) than FPD (80.3%: 78.7 to 81.9%). It cost four times less to conduct an HIV test using SFs ($3.90 per SF HIV test) than FPD ($13.98) and five times less to link a client to care with SFs ($62.74) than FPD ($303.13).

**Conclusions:**

SF HTS was effective in identifying more clients, first‐time HIV testers and more HIV‐positive people, but less effective in linking clients to care than FPD HTS. The SF HTS model was cheaper than the FPD‐employee model. We recommend strengthening SFs particularly their linkage to care activities.

## 1 Introduction

With an estimated 7.1 million people living with HIV (PLHIV) and almost 270,000 people newly infected with HIV in 2016 1, 2, 3 South Africa has a large HIV burden that requires urgent attention. While 14% remain undiagnosed, 44% unlinked to HIV care and treatment and 54% not virally suppressed 4 four years before the UN's 90‐90‐90 goals are expected to be reached, South Africa urgently needs a comprehensive and innovative HIV testing and linkage programme. The traditional voluntary counselling and testing in health facilities achieved low uptake of HIV testing services (HTS) due to health system‐related factors (long travel distance, long waiting times, inflexible service hours, staff attitudes, privacy) and patient‐related factors (health‐seeking behaviours, negative attitudes towards HTS, fear of rejection or discrimination) 5, 6, 7. This led to the launch of the community‐based HIV counselling and testing (CBCT) programme to increase coverage, uptake and linkage to care run by non‐governmental players among others. Still, new and innovative ways to increase coverage, positivity and linkage to care need to be tested.

The Programme of Action of the International Conference on Population and Development, held in Cairo in 1994 8, challenged countries to consider public‐private partnerships to achieve health goals. This resolution was revisited by the Universal Access to Health for All by year 2015 9. A number of strategies to achieve health for all have since been tried, including privatization of health services. One of the privatization concepts being tested globally is social franchising. Franchising is a concept from the commercial industry in which a franchisor, usually a large business, contracts a franchisee, usually a small business, to produce, market or provide its successful service or brand according to a blueprint developed or owned by the franchisor 10. The goal of social franchising is to offer a better service than the available service at a low price with increased availability through the use of a commercial relationship between the franchisor and franchisee. The franchisor defines a product and brand, delivery mode, quality assurance, quality standards and provides a brand and staff training 11.

Few studies have assessed franchised reproductive health and family planning services delivered through community‐based public health platforms. The social franchising model has largely been applied to increase coverage, affordability and effectiveness of family planning services 12, 13, 14, and of late has also been extended to expand TB testing 11, access to general practitioner consulting 15, improving maternal health 16 and child health 17. Research indicates that franchising of private reproductive health and family planning services is associated with improved service provision, quality and utilization in low‐ and middle‐income contexts 18, 19, 20, 21. Despite the effectiveness of social franchising to reproductive health and family planning programmes, enormous challenges have been cited in a comprehensive review of 45 clinical social franchises in 27 countries across Africa, Asia and Latin America 22 as: non‐improving coverage, unsustainable or unaffordable services, no evidence of adherence to quality protocols, difficulties in recruiting franchisees, high franchisee attrition, franchisees’ inability to attend training programmes, use of untrained lay health workers to deliver services without support or supervision, and logistical problems in using quality assurance tools. The authors concluded that continued investment in social franchising of health programmes requires new evidence of their importance and utility 22. However, none of these 45 studies focused on HTS, which our study focuses on.

During the 2000s, many studies evaluated the feasibility of lay counsellors in conducting CBCT and have shown that it is feasible to employ this cadre of employees 23, 24, 25, 26, 27, 28. However, the feasibility of a counsellor‐led‐and‐managed business in HIV counselling, testing and linkage to care (as compared to businesses managed by public or private health organizations in which counsellors are employees) has not been tested in CBCT programmes. We therefore developed, tested and evaluated a pilot study of a SF CBCT programme and compared it with an existing FPD CBCT programme as our control. We analysed routine programme data to assess the effectiveness of the SF model in testing clients, identifying HIV‐positive clients and linking them to HIV care programmes in health facilities.

## 2 Methods

### 2.1 Study setting

Tshwane is the second largest district in Gauteng province. Over a tenth (12.6%) of the people living in Gauteng province are HIV positive and this is at par with the national prevalence 1. Close to one in five 15‐ to 49‐year olds is infected 1. About 77,000 new infections were reported between 2015 and 2016, an incidence rate of 0.98%, slightly lower than the national incidence rate of 1.05%. The province accounts for a third (34.2%) of all annual HIV deaths in the country. About 75% of all PLHIV in Gauteng province know their status, but only 55% are on treatment of which only 46.4% are virally suppressed illustrating that the province falls behind in all the three UN's 90‐90‐90 HIV targets 1. The province is characterized with high levels of ethnic and socio‐economic heterogeneity.

### 2.2 Programme design and implementation

#### 2.2.1 Foundation for professional development HIV testing services

FPD implemented a CBCT programme to identify PLHIV and link them to HIV care and treatment 29 to contribute towards the UNAIDS's 90‐90‐90 goal 30 while aligning with the goals and targets of the South African Government's National Strategic Plan on HIV, STIs and TB (2012 to 2016) 31 as well as the PEPFAR/SA HIV Prevention Strategy 32. The CBCT programme mainly utilized the home‐based approach to recruit participants. FPD formally advertised, employed and trained counsellors to conduct HTS and managed their daily HTS activities while paying them monthly remunerations that were not necessarily pegged according to HTS outputs. Table 1 illustrates the main components of the FPD and SF activities.

**Table 1:** Process flow for the FPD and social franchise HTS

Item	Activity	FPD	Social franchise
1 Counsellor Recruitment and contracting	Contract	1. Employed by FPD 2. Normal employment contract 3. Normal payroll contract 4. Work for eight hours a day	1. Unemployed, self‐employed or employed elsewhere, not employed by FPD 2. Contracted by franchisee‐franchisor contract 3. No restriction on hours, days worked 4. Test ≥100 people per month 5. Test >/5 HIV + tests per month 6. Link ≥4 HIV + clients per month
	Remuneration	1. Fixed monthly salary	1. Paid $3.20 per HIV test 2. Paid $8 per HIV + client linked
	Training	Training focused on: 1. Pre‐test, test, post‐test, documentation, referral and linkage to care	Training focused on: 1. Pre‐test, test, post‐test, documentation, referral and linkage to care 2. Business development and management
2 Mobilization	Community level mobilization	Community events; flyers; campaigns (e.g. couples counselling); conducting systematic door‐to‐door mobilization campaigns distributing flyers, and condoms	Community events; flyers; campaigns (e.g. couples counselling); conducting systematic door‐to‐door mobilization campaigns distributing flyers, and condoms
	Individual level mobilization	Informing households on the benefits and availability of testing for HIV; scheduling household level testing appointments and/or actively recruiting clients for same‐day HTS	Informing households on the benefits and availability of testing for HIV; scheduling household level testing appointments and/or actively recruiting clients for same‐day HTS
3 Documentation	Questionnaire and database	Questionnaire interview on: 1. Demographic characteristics 2. HIV testing history 3. HIV test results 4. Referral 5. Linkage results 6.(bi)monthly, quarterly, annual reports	Questionnaire interview on: 1. Demographic characteristics 2. HIV testing history 3. HIV test results 4. Referral 5. Linkage results 6. (bi)monthly, quarterly, annual reports
	Payment	Paid per normal payroll	Monthly payment claims to franchisor
4 Basic package of services	Pre‐test	1. Information and education 2. Screening 3. Condom education and distribution	1. Information and education 2. Screening 3. Condom education and distribution
	Test	1. Informed consent 2. HIV testing using national algorithm	1. Informed consent 2. HIV testing using national algorithm
	Post‐test	1. Tailor‐made counselling based on test result(s) and life situation 2. Referral for psychosocial support 3. Referral for HIV treatment and care 4. Development of tailored linkage plans and timelines looking at individual barriers and concerns 5. If negative: risk reduction counselling, appropriate HIV prevention services	1. Tailor‐made counselling based on test result(s) and life situation 2. Referral for psychosocial support 3. Referral for HIV treatment and care 4. Development of tailored linkage plans and timelines looking at individual barriers and concerns 5. If negative: risk reduction counselling, appropriate HIV prevention services
5 Follow‐up	Referral to HIV care and treatment	Discuss referral health facility	Discuss referral facility
	Linkage and service update	Actively follow‐up to verify and document linkage	Actively follow up to verify and document linkage

FPD, Foundation for Professional Development; HTS, HIV testing services.

#### 2.2.2 Social franchise HIV testing services or SFs

In 2016, FPD introduced the Social franchise testing model 33 into the existing CBCT approaches (see Table 1). The programme was an effectiveness community‐based HTS study implemented through SFs between October 2016 and September 2017 in Tshwane district, South Africa to provide evidence of success or failure for community rollout in a sustainable public‐private partnership context. We advertised and encouraged small‐scale businesses, employed or unemployed individuals to apply for the SF opportunity. Successful candidates were selected through an interview process and provided a 10‐day training on HTS, documentation, and referral for antiretroviral therapy. In addition, SFs received training on small business management to equip them with relevant skills in business management. SFs delivered standards‐based HTS (recruit clients from the community, test them and link HIV‐positive clients to referral health facilities) under a common brand implemented by FPD while FPD systematized and prescribed standards for HTS accreditation and ongoing quality assurance of the SFs.^1^ The SFs were paid R40 (US$3.20) per HIV test conducted including, providing a basic package of services to each client tested (such as condom distribution, screening for tuberculosis (TB) and sexually transmitted infection) and documenting the client. A much bigger remuneration of R100 (US$8) was paid for every HIV‐positive client linked to motivate the more difficult processes of linking HIV‐positive clients into care. The minimum contractual requirements for SFs included testing at least 100 people per month, finding five HIV‐positive individuals and successfully linking at least four of them to care. FPD monitored programme performance data, HIV testing and referral process activities of SFs through monthly, bi‐monthly and quarterly reports, field visits and observations. FPD CBCT programme managers conducted field visits and observations to support SF HTS services and check compliance with CBCT guidelines as well as addressing challenges faced such as in linking clients to care at health facilities.

### 2.3 Data collection and analysis

A questionnaire was developed and set up on a personal digital assistant (PDA) to enter all data collected from the client. Demographic, HIV testing history, couple or individual testing, HIV results and linkage to care including referral data were captured onto the PDA through a one‐on‐one counsellor‐administered interview with each client enrolled for HTS. In cases where the PDA fails to work, for example, battery runs out, paper‐based questionnaires were completed and were later captured electronically and stored onto the study database. Linkage to care information obtained from health facilities were captured onto the database as well. Four primary outcomes were used to assess programme success and these are HIV testing proportions, HIV positivity, linkage to care and counsellor cost. Costing data were obtained from counsellor/SFs remuneration records. These indicators were documented on electronic and/or paper‐based forms. *HIV testing proportion* was calculated as all clients counselled and tested for HIV in each study group including being offered a basic package of HTS divided by the total CBCT clients tested multiplied by 100. *HIV positivity* was calculated as the proportion of clients who tested HIV positive divided by the total number of clients tested for HIV. *Linkage to HIV care and treatment rate* was assessed as a proportion of all clients who tested positive during the current reporting 12‐month period and were successfully linked to care within the same period divided by the number of all clients who tested HIV positive during the same reporting period. *Counsellors’ remuneration cost* per person tested, HIV‐positive test and person linked to care were calculated to determine the mean cost of employing counsellors in each study group. We also assessed secondary outcome indicators. We calculated the average counsellor remuneration cost incurred to test each client, to identify an HIV‐positive client and link each positive test to care per study group, for example, the counsellor cost per HIV‐positive test was found by dividing the total amount of money spent on each group to pay all counsellors by the total number of HIV‐positive tests conducted. The costing calculation excluded the remuneration of project management staff such as Monitoring and Evaluation officers. *First‐time HIV testers* rate was defined as the proportion of persons identified as testing for HIV for the first time divided by the total number of people tested in a given period. *Couple HIV testing* rate was defined as the proportion of two people identifying themselves as partners who tested and received results together divided by the total number of clients testing. Available programme quarterly and monthly reports were reviewed to obtain more information on the implementation of the two models. The analysis of the primary outcomes involved comparing HIV testing proportions, positivity, linkage to HIV care and treatment and counsellor cost in the two study groups: FPD HTS and SFHTS. Descriptive statistics were used to describe the data. Proportions stratified by age group and gender were calculated. The 95% CIs were used to compare proportions between FPD and SF. All analyses were carried out using STATA 13.0.

### 2.4 Ethics

The study received ethics approval from the FPD Research Ethics Committee. The Department of Health gave permission to implement the CBCT programme. Clients provided informed written consent to participate in the programme while guardians/parents consented for children under 12. The trial was registered with the Pan African Clinical Trial Registry (PACTR201809873079121).

## 3 Results

### 3.1 Human resources

Table 2 shows the distribution of counsellors for HTS by study group. In total, 43 counsellors conducted HTS during the course of the study. The SFs were 19 (44.2%) while FPD had 24 employees (55.8%), whose total time on the project was equivalent to 19.9 full time counsellors making the number of counsellors similar to those in the SF group. There were slightly more female counsellors in the SF than in the FPD group (94.8% vs. 75%). The median age of counsellors was 38 years (interquartile range (IQR) 32.2 to 43.6); 39 years (IQR 33.0 to 43.2) in the SF and 38 years (IQR 31.7 to 43.2) in the FPD group. A higher proportion of FPD counsellors were more educated to tertiary levels than SFs (70.8% vs. 21.1%). Counsellors’ work experience at the beginning of the pilot did not differ by study group. While all FPD employees worked primarily during weekdays between 07:30 and 16:30 hours SFs independently determined their time of work and they tended to extend working hours and worked during weekends and public holidays.

**Table 2:** HIV counsellors’ demographic characteristics by study group

Variable	Total N (%)	Social franchisees N (%)	FPD employees N (%)^a^
Counsellors	43 (100)	19 (44.2)	24 (55.8)
Gender			
Male	7 (16.3)	1 (5.3)	6 (25)
Female	36 (83.7)	18 (94.7)	18 (75.0)
Age (years)			
25 to 34	15 (34.9)	6 (31.6)	9 (37.5)
35 to 44	20 (46.5)	9 (47.4)	11 (45.8)
45 to 54	3 (7.0)	3 (15.8)	0 (0.0)
55 to 64	3 (7.0)	0 (0.0)	3 (12.5)
65+	2 (4.7)	1 (5.3)	1 (4.2)
Highest level of education			
No matric	4 (9.3)	4 (21.1)	0 (0)
Matric	18 (41.9)	11 (57.9)	7 (29.2)
Tertiary	21 (48.8)	4 (21.1)	17 (70.8)
Work experience			
Yes	34 (79.1)	15 (78.9)	19 (79.2)
No	9 (20.9)	4 (21.1)	5 (20.8)
Total	43 (100)	19 (100)	24 (100)

FPD, Foundation for Professional Development; SF, social franchisee.

a Although 24 counsellors were employed by FPD during this time, 19.9 full‐time equivalent employees worked on this project making the number of employees similar to those in the SF arm.

### 3.2 HIV testing proportions

**Figure 1:**
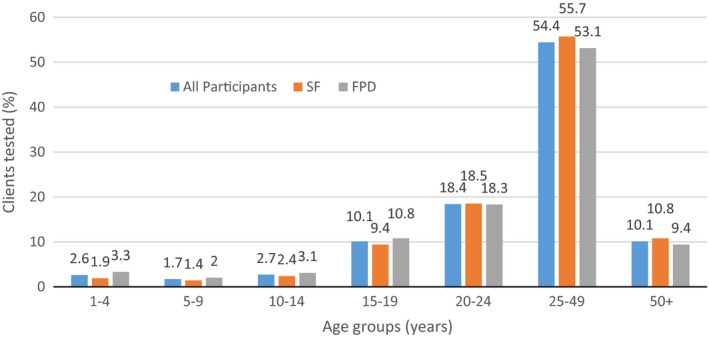
HIV testing rates.

Table 3 shows demographic characteristics of clients tested by study group while Figure 1 shows total clients tested in the FPD or SF arm by age group. A total of 84,556 individuals tested for HIV with slightly more participants tested by SFs (50.5%: 50.2 to 50.8) than by FPD HTS (49.5%: 49.2 to 49.8). Slightly more females (51.2%: 50.8 to 51.5) than males (48.8%: 48.5 to 49.2) tested overall but SFs tested more females (54.1%: 53.6 to 54.6) than males (48.2%: 47.7 to 48.7) compared to FPD HTS. Over 90% of those who tested were 15 years or older in each group. SFs tested less proportions of children (1 to 14) overall and female and male children than FPD employees. There were no differences in HIV testing by gender among clients aged 20 to 24 years in the two groups. Among males, the proportion of young men in the age group 20 to 24 years who tested was almost double the proportion of the adolescents in the 15 to 19 years age group which was also double the proportion of males in the 10 to 14 which in turn was similarly twice that of the five‐ to nine‐year age group. Among females, the proportion of clients in the 20 to 24 years who tested was double the proportion of those tested in the 15‐ to 19‐year age group in each study group. The SFs identified more first‐time testers (21.5%: 21.1 to 21.9) than FPD employees (8.9%: 8.6 to 9.1). Only 2.6% of the individuals tested as couples with a higher proportion of couples tested by FPD employees (4.6%: CI 4.4 to 4.8) than by SFs (0.6%: CI 0.5 to 0.7).

**Table 3:** Uptake of HTS by study group and client demographic characteristics

Variable	Total N (%)	Social franchisee HTS N (%)	95% CI	FPD employee HTS N (%)	95% CI
Total tested	84,556 (100)	42,697 (50.5)		41,859 (49.5)	
Gender					
Male	41,280 (48.8)	19,607 (45.9)	45.4 to 46.4	21,673 (51.8)	51.3 to 52.3
Female	43,276 (51.2)	23,090 (54.1)	53.6 to 54.6	20,186 (48.2)	47.7 to 48.7
Total	84,556 (100)	42,697 (100)		41,859 (100)	
Age					
One to four years	2177 (2.6)	800 (1.9)	1.7 to 2.0	1377 (3.3)	3.1 to 3.5
Five to nine years	1449 (1.7)	601 (1.4)	1.3 to 1.5	848 (2.0)	1.9 to 2.2
10 to 14 years	2322 (2.7)	1014 (2.4)	2.2 to 2.5	1308 (3.1)	3.0 to 3.3
15 to 19 years	8525 (10.1)	4000 (9.4)	9.1 to 9.6	4525 (10.8)	10.6 to 11.1
20 to 24 years	15,537 (18.4)	7890 (18.5)	18.1 to 18.9	7647 (18.3)	17.9 to 18.6
25 to 49 years	46,031 (54.4)	23,799 (55.7)	55.3 to 56.2	22,232 (53.1)	52.6 to 53.6
50+ years	8515 (10.1)	4593 (10.8)	10.5 to 11.1	3922 (9.4)	9.1 to 9.7
Total	84,556 (100)	42,697 (100)		41,859 (100)	
Males					
One to four years	1064 (2.6)	385 (2.0)	1.8 to 2.2	679 (3.1)	2.9 to 3.4
Five to nine years	708 (1.7)	305 (1.6)	1.4 to 1.7	403 (1.9)	1.7 to 2.0
10 to 14 years	1309 (3.2)	623 (3.2)	2.9 to 3.4	686 (3.2)	2.9 to 3.4
15 to 19 years	3650 (8.8)	1615 (8.2)	7.9 to 8.6	2035 (9.4)	9.0 to 9.8
20 to 24 years	6607 (16.0)	3116 (15.9)	15.4 to 16.4	3491 (16.1)	15.6 to 16.6
25 to 49 years	23,740 (57.5)	11,427 (58.3)	57.6 to 59.0	12,313 (56.8)	56.1 to 57.5
50+ years	4202 (10.2)	2136 (10.9)	10.5 to 11.3	2066 (9.5)	9.1 to 9.9
Total	41,280 (100)	19,607 (100)		21,673 (100)	
Females					
One to four years	1113 (2.6)	415 (1.80)	1.6 to 2.0	698 (3.46)	3.2 to 3.7
Five to nine years	741 (1.7)	296 (1.28)	1.1 to 1.4	445 (2.20)	2.0 to 2.4
10 to 14 years	1013 (2.3)	391 (1.69)	1.5 to 1.9	622 (3.08)	2.8 to 3.3
15 to 19 years	4875 (11.3)	2385 (10.33)	9.9 to 10.7	2490 (12.34)	11.9 to 12.8
20 to 24 years	8930 (20.6)	4774 (20.68)	20.2 to 21.2	4156 (20.59)	20.0 to 21.2
25 to 49 years	22,291 (51.5)	12,372 (53.58)	52.9 to 54.2	9919 (49.14)	48.4 to 49.8
50+ years	4313 (10.0)	2457 (10.64)	10.2 to 11.0	1856 (9.19)	8.8 to 9.6
Total	43,276 (100)	23,090 (100)		20,186 (100)	
Uptake of HIV testing and counselling					
First‐time testers	12,900 (15.3)	9189 (21.5)	21.1 to 21.9	3711 (8.9)	8.6 to 9.1
Repeat testers	71,656 (84.7)	33,508 (78.5)	78.1 to 78.9	38,148 (91.1)	90.8 to 91.4
Total	84,556 (100)	42,697 (100)		41,859 (100)	
Couple testing					
Tested as couples	2200 (2.6)	264 (0.6	0.5 to 0.7	1936 (4.6)	4.4 to 4.8
Tested as individuals	82,356 (97.4)	42,433 (99.4)	99.3 to 99.5	39,923 (95.4)	95.2 to 95.6
Total	84,556 (100)	42,697 (100)		41,859 (100)	

FPD, Foundation for Professional Development; HTS, HIV testing services.

### 3.3 HIV positivity

**Figure 2:**
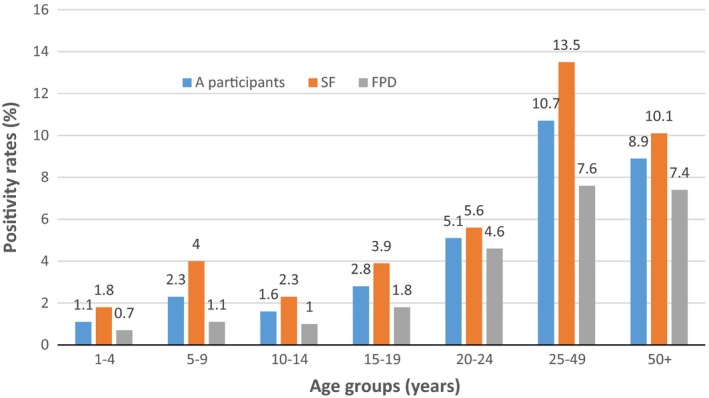
HIV positivity rates.

Table 4 shows HIV positivity rate by study group while Figure 2 shows the positivity rate by age group. In the total sample, 8%: 7.9 to 8.2 of the HTS clients tested HIV positive. More HIV‐positive participants were identified/diagnosed by SFs (10.2%: 9.9 to 10.5) than by FPD employees (5.9%: 5.6 to 6.1). Both study groups found higher HIV positivity among females than males. Sharp HIV positivity differences were observed by age group. The SFs identified higher positivity (13.5%: 13.1 to 14.0) in the 25 to 49 year age group compared to FPD (7.6%: 7.3 to 8.0) while FPD found a lower positivity in the 50+ years age group (7.4%: 6.6 to 8.3) compared to SF's (10.1%: 9.3 to 11.0), followed by the 20 to 24 year age group (FPD 4.6%: 4.2 to 5.1 vs. SFs 5.6%: 5.1 to 6.2). Among males aged 50 years and above, SFs tested 9.6%: 9.4 to 11.9 HIV‐positive individuals compared to 6.8%: 5.8 to 8.0 tested by FPD employees. Similar rates of positivity were found among the younger age groups, while positivity among the under 10s was much higher in SF group than in the FPD group in the male population. Among females, HIV positivity differences by age groups were observed in all age groups with higher positivity found by SFs than by FPD employees.

**Table 4:** HIV positivity and linkage to care by trial arm

Variable	Total N (%)	Social franchisee HTS		FPD employee HTS	
		N (%)	95% CI	N (%)	95% CI
HIV positivity					
HIV positivity	6795 (8.0)	4342 (10.2)	9.9 to 10.5	2453 (5.9)	5.6 to 6.1
HIV negativity	77,761 (92.0)	38,355 (89.8)	89.5 to 90.1	39,406 (94.1)	93.9 to 94.4
Total sample	84,556 (100.0)	42,697 (100.0)		41,859 (100.0)	
HIV positivity by gender					
Male	2907 (7.0)	1770 (9.0)	8.6 to 9.4	1137 (5.2)	5.0 to 5.6
Female	3888 (9.0)	2572 (11.1)	10.7 to 11.6	1316 (6.5)	6.2 to 6.9
Total	6795 (8.0)	4342 (10.2)	9.9 to 10.5	2453 (5.9)	5.6 to 6.1
HIV positivity by age					
One to four years	23 (1.1)	14 (1.8)	1.0 to 2.9	9 (0.7)	0.3 to 1.2
Five to nine years	33 (2.3)	24 (4.0)	2.6 to 5.9	9 (1.1)	0.5 to 2.0
10 to 14 years	36 (1.6)	23 (2.3)	1.4 to 3.4	13 (1.0)	0.5 to 1.7
15 to 19 years	237 (2.8)	156 (3.9)	3.3 to 4.5	81 (1.8)	1.4 to 2.2
20 to 24 years	797 (5.1)	444 (5.6)	5.1 to 6.2	353 (4.6)	4.2 to 5.1
25 to 49 years	4912 (10.7)	3216 (13.5)	13.1 to 14.0	1696 (7.6)	7.3 to 8.0
50+ years	757 (8.9)	465 (10.1)	9.3 to 11.0	292 (7.4)	6.6 to 8.3
Total	6795 (8.0)	4342 (10.2)	9.9 to 10.5	2453 (5.9)	5.6 to 6.1
HIV positivity by age group and gender					
Males					
One to four years	11 (1.0)	8 (2.1)	0.9 to 4.1	3 (0.4)	0.1 to 1.3
Five to nine years	20 (2.8)	14 (4.6)	2.5 to 7.6	6 (1.5)	0.5 to 3.2
10 to 14 years	20 (1.5)	13 (2.1)	1.1 to 3.5	7 (1.0)	0.4 to 2.1
15 to 19 years	53 (1.5)	33 (2.0)	1.4 to 2.9	20 (1.0)	0.6 to 1.5
20 to 24 years	235 (3.6)	116 (3.7)	3.1 to 4.4	119 (3.4)	2.8 to 4.1
25 to 49 years	2223 (9.4)	1382 (12.1)	11.5 to 12.7	841 (6.8)	6.4 to 7.3
50+ years	345 (8.2)	204 (9.6)	8.3 to 10.9	141 (6.8)	5.8 to 8.0
Total	2907 (7.0)	1770 (9.0)	8.6 to 9.4	1137 (5.2)	5.0 to 5.6
Females					
One to four years	12 (1.1)	6 (1.4)	0.5 to 3.1	6 (0.9)	0.3 to 1.9
Five to nine years	13 (1.8)	10 (3.4)	1.6 to 6.1	3 (0.7)	0.1 to 2.0
10 to 14 years	16 (1.6)	10 (2.6)	1.2 to 4.7	6 (1.0)	0.3 to 2.1
15 to 19 years	184 (3.8)	123 (5.2)	4.3 to 6.1	61 (2.4)	1.9 to 3.1
20 to 24 years	562 (6.3)	328 (6.9)	6.2 to 7.6	234 (5.6)	4.9 to 6.4
25 to 49 years	2689 (12.1)	1834 (14.8)	14.2 to 15.5	855 (8.6)	1.1 to 1.3
50+ years	412 (9.6)	261 (10.6)	9.4 to 11.9	151 (8.1)	6.9 to 9.5
Total	3888 (9.0)	2572 (11.1)	10.7 to 11.6	1316 (6.5)	6.2 to 6.9
Linkage to care					
Cumulative linkage to care	4575 (67.3)	2605 (60.0)	58.5 to 61.5	1970 (80.3)	78.7 to 81.9

CI, confidence interval; FPD, Foundation for Professional Development; HTS, HIV testing services.

### 3.4 Linkage to HIV care and treatment

Linkage to care results is shown in Table 4. A total of 67.2% were linked to HIV care and treatment during the study period. Of these, SFs linked 60% (58.5 to 61.5) while FPD employees linked 80%: 78.7% to 81.9% to care. From the quarterly and monthly reports we found that SFs reported linkage to care challenges including not being accepted at health facilities by health staff when they wanted to check their clients’ linkage to care details. These challenges were addressed but still resurfaced.

### 3.5 HIV counsellor expenditures

Table 5 shows HIV counsellor cumulative average salary expenditures for the 2016 to 2017 by clients tested, HIV positivity and clients linked to care. Both the SF and the FPD models had similar populations to test for HIV in the same district although their offers to test were accepted differently by different population groups as described earlier. Overall, the SF model was significantly more cost effective to test, identify HIV‐positive people and link them to care compared to FPD employees (R5 m vs. R1.9 m). The total HIV counsellor cost of conducting an HIV test was calculated to be R174.80 per FPD employee and R48.80 per SFs. This makes conducting HIV testing through the SFs more than three and a half times cheaper than through FPD employees. The cost of identifying HIV positive clients using FPD employees was R3032.20 per test while it only cost SFs R470.50 per test. SFs were therefore 6.4 times cheaper than using FPD employees. It was almost five times cheaper to link HIV‐positive individuals to HIV care and treatment through SFs (R784.20 per person linked) than FPD employees (R3789.10).

**Table 5:** Costing of HIV counsellors per HTS, positivity and linkage to care by study group

Variable	Total (N)	Social franchisee HTS	FPD employee HTS
		Cost (Rand) (%)	Cost (Rand) (%)
Total spent (R million)	9.44	2.04 (21.6)	7.4 (78.4)
Remuneration (R million)	6.9	1.9 (27.5)	5.0 (72.5)
Other expenses (R million)	2.54	0.14 (5.5)	2.4 (94.5)
Cost per individual tested (R)	223.6	48.8 (21.8)	174.8 (78.2)
Cost per HIV test (R)	3502.7	470.5 (13.4)	3032.2 (86.6)
Cost per individual linked to care (R)	4573.3	784.2 (17.1)	3789.1 (82.9)

Currency exchange rate: 1US$=R12.5. FPD, Foundation for Professional Development; HTS, HIV testing services.

## 4 Discussion

The objective of the study was to test the effectiveness of the social franchise model in creating demand for HIV test, identify HIV‐positive people and link them to care. It also aimed at estimating the counsellor salary cost required to create demand, find HIV‐positive people and link them to care. The study found that more SFs were less educated and had less basic remuneration than FPD counsellors but effectively tested slightly more clients, identified more HIV‐positive people than FPD counsellors. An unexpected result was that SFs linked fewer clients to care than FPD employees. Finally, results show that SFs were more affordable than FPD employees as they tested more people, identified more HIV‐positive people and linked them to care at less per counsellor cost compared to FPD employees. To the best of our knowledge, this is the first community‐based HTS SF model and one that shows the effectiveness of a counsellor‐led and managed CBCT programme. We discuss the implications and meaning of these results below.

The SF model created jobs, offered training and expertise at no cost to largely unemployed and self‐employed people as SFs and offered them opportunities to formally link with formal organizations including government institutions. Considering that SFs were generally less educated than FPD counsellors, such training helped to empower community health workers and strengthen the country's health system even beyond the lifespan of this project. This capacity building programme increased SFs’ employability and business networks for further and more meaningful collaborative work with both the government and non‐governmental organizations. The study not only adds to the literature on the importance of community health workers 34, 35 but also on how innovative HIV prevention programmes can include community members in more useful and effective ways in view of limited resources for HIV prevention and treatment. This paper highlights community health workers’ contribution and recognition.

The fact that SFs were remunerated on commission may have motivated the SF counsellors to achieve higher rates of HIV testing and HIV positivity compared to FPD employees who worked on a business‐as‐usual mode with less motivation to increase productivity as their salaries did not change. Results show the feasibility and affordability of a results‐based remuneration system which is target oriented and self‐motivating in community based work. Our study provides evidence of increased access to HTS using SFs at reduced human resources costs. Previous research found that SFs increase the public's access to services 11, 12, 18, 21, 36 although this was not with HTS which we therefore provide knew knowledge for. Working longer hours more than FPD employees and living in communities they worked made it feasible for SFs to test more clients with fewer counsellors and less remuneration. That more first‐time testers would be reached by SFs than with FPD employees could also be a result of the SFs’ good knowledge of where those who never tested were found and their ability to create demand among fellow community members. Our findings provide evidence for the need to scale up SF HTS to cover the 90‐90‐90 gaps for South Africa. Because FPD is a recognized organization, their counsellors were able to organize campaigns for couple testing compared to the less recognized and independently operating SFs who needed more support in this activity.

The low uptake of HIV testing among men in SFs confirms previous trends 37 which have resulted in incentivising men to increase HIV testing rates 38. Although we understand that females have better health seeking behaviours than men further analysis is needed to assess if the gender of the counsellor influences uptake since there was a significantly higher proportion of female than male counsellors in the SF than in the FPD model where more females tested. A demand creation strategy is required to convince those who never tested before to test until saturation point is reached.

It is important to unpack the possible reasons why SF had higher HIV positivity rates for the individuals they tested for HIV compared to what was obtained by FPD employees. First, having a knowledge of the hotspots helps to target HIV testing better for higher yield 39, 40. Because SFs lived in the communities where they operated, they had knowledge of the HIV hotspots compared to FPD employees who “commuted” into the area for work purposes and returned to the FPD office within an eight hour working day. Second, identifying HIV‐positive clients was a step closer to earning a relatively large sum of money once a client is successfully linked to care and this remuneration might have motivated SFs to increase the efforts to find the HIV‐positive clients. In addition, challenges to link clients which resulted in not earning much from linkage to care might have motivated SFs to test more clients with the hope that at least some of them will be positive and would be linked to care successfully.

We found that SFs linked less clients to HIV care and treatment than FPD employees. SFs faced challenges of access to client data at the health facilities and this was noted during the pilot monitoring meetings. Despite having introduced the SFs to the health facility management to enable them to access facility records to collect data on linkage to care, there still remained some gate keepers in some facilities who mistrusted SFs as they were not directly employed by FPD or any recognized organization. On the other hand, FPD employees had a coordinated linkage to care programme in which all counsellors shared the testing and client data, which individual SFs did not do. Each SF worked as an individual entity and had to visit every facility where their clients were referred for care which may have frustrated facility gatekeepers. Similar logistical challenges have been reported in a review of social franchises 22 while challenges in connecting clients to the clinics 41 and actual client's healthcare utilization 21, 41 have been reported in SFs before. FPD is currently developing software integrations with the National Health Laboratory Services (NHLS) and TIER.net national and electronic patient management system to improve data access and verification to ease linkage to care processes. It is hoped that this will help address SF challenges.

The paper has some limitations. First, it is based on routinely collected programme data. The data elements were not designed to answer our study objectives. Studies of the South African health information system have shown that routinely collected data are usually incomplete and inaccurate to a greater extent 42, 43 rendering some variables and data unusable for policy and practice. Our data were affected by incompleteness in some variables which we had to drop from the analysis. For example, we do not know how many people were offered but refused HIV testing in each study arm. The database only included data from clients who accepted HIV testing. Information about refusals to test could have been useful in establishing the acceptability of each model. Second, our data were disaggregated for programmatic purposes, for example, the variable age was in accordance with donor reporting requirements which put more emphasis on adults aged 25 to 49 years as the highest risk age group and yet categorizing adults into this one large stratum blurs existing differences in behaviours, uptake, and linkage to care. Third, the data did not allow conducting individual level analysis which could have enabled different analyses including for example uptake or linkage by individual counsellor or client characteristics. Despite these limitations the study has its strengths, which include a large sample size drawn from three sub districts in Tshwane, making generalization to the Tshwane district possible. The pilot shows promising results to motivate the implementation of the SF model and that the costing analysis informs the importance of remunerating employees based on performance compared to monthly remuneration that is not based on set targets. We were able to demonstrate that it is cheaper to use SF counsellors than to employ full time counsellors under FPD for CBCT work.

## 5 Conclusions

We were able to demonstrate through this pilot study that the social franchise model can test more people, identify more HIV‐positive people and first‐time testers at reduced per counsellor cost, but that it is less effective in linking clients to HIV care and treatment than the full time FPD employee model. These encouraging results led FPD to expand the SFHTS project to other districts, while addressing the challenges identified in the study, such as automating the linkage to care process through database integrations between the FPD CBCT testing application, used by all franchisees, and the NHLS and TIER.net systems. FPD will make the use of its electronic testing application a requirement for the SF HTS model. This system is customised for the SF HTS model to remunerate testing based on the HTS, positivity and linkage data completed on the App. Franchisee remuneration will thus be automated, ensuring good governance, control and audit compliance. A lay counsellor‐led and managed CBCT programme can save money while reaching more participants through the SF model. The study also adds to existing literature more broadly that private SFs can more effectively deliver CBCT.

## Competing interest

The funders had no role in study design, data collection and analysis, decision to publish, or preparation of the manuscript.

## Authors’ contributions

SS conceived and designed the study and led the data collection, analysis and interpretation of data, drafted the article, led the revisions and approved the version to be published. NN, NM, JK, TF, LK, SJ, JS and GG substantially contributed towards study design, data analysis, and interpretation of data, revision of the manuscript and approved the final version to be published.

## Note

1 FPD duties and support for SFs included assisting to establish linkages between SFs and the referral health facilities (particularly to access HTC commodities and waste disposal services and support in‐facility linkage and verification exercises); providing access to IEC and marketing material; facilitating coordination with local mobilization strategies; and providing mentorship and support around management of small business skills development.
